# Improved Line Tracing Methods for Removal of Bad Streaks Noise in CCD Line Array Image—A Case Study with GF-1 Images

**DOI:** 10.3390/s17040935

**Published:** 2017-04-24

**Authors:** Bo Wang, Jianwei Bao, Shikui Wang, Houjun Wang, Qinghong Sheng

**Affiliations:** 1 College of Astronautics, Nanjing University of Aeronautics and Astronautics, Nanjing 210016, China; baojianwei_nuaa@163.com (J.B.); qhsheng@nuaa.edu.cn (Q.S.); 2 Tianjin Key Laboratory of Intelligent Information Processing in Remote Sensing, Tianjin 300301, China; wangshk@spacestar.com.cn; 3 National Ocean Technology Center, Tianjin 300112, China; whjdn@163.com

**Keywords:** bad streaks noise, line tracing method, DN values break, Lagrange polynomial method, DN values succession

## Abstract

Remote sensing images could provide us with tremendous quantities of large-scale information. Noise artifacts (stripes), however, made the images inappropriate for vitalization and batch process. An effective restoration method would make images ready for further analysis. In this paper, a new method is proposed to correct the stripes and bad abnormal pixels in charge-coupled device (CCD) linear array images. The method involved a line tracing method, limiting the location of noise to a rectangular region, and corrected abnormal pixels with the Lagrange polynomial algorithm. The proposed detection and restoration method were applied to Gaofen-1 satellite (GF-1) images, and the performance of this method was evaluated by omission ratio and false detection ratio, which reached 0.6% and 0%, respectively. This method saved 55.9% of the time, compared with traditional method.

## 1. Introduction

The Gaofen-1 satellite (GF-1) was launched on 6 April, 2013, with sensors that include two meter full-color Charge-Coupled Device (CCD) camera, eight-meter multi-spectral CCD camera and 16 m multi-spectral CCD camera. The data width of the two meter full-color CCD camera and eight meter multi-spectral CCD camera is better than 60 km, and the data is stitched by two cameras. The data width of the 16 m multi-spectral CCD camera is better than 800 km, stitched by four cameras. These high-resolution remote sensing images are widely used in agricultural and water resources surveys [1].

In these remote sensing image applications, the most important data is the remote sensing image pixel brightness value, also known as DN value. However, due to the difference in the CCD cameras, or the tremor of the platform [2], the image will lose some data, and pixel DN value will suddenly become zero. These pixels will form random stripes in the image, and these stripes are the bad streaks noise described in this paper. Bad streaks are only approximately five pixels wide, but the length can reach 1/3 of the entire image. According to the incomplete record of the data obtained by Suzhou Tianqi Company for 2015–2016, 20% of the images are influenced by bad streaks noise. Since the DN value of the bad line area becomes zero, image entropy will be reduced, and bad streaks may even be misjudged as a linear target, resulting in an image matching error. Bad streaks noise makes the images inappropriate for vitalization and batch process, and it is necessary to find a method to satisfactorily deal with this noise.

Previous studies show that moment matching and Fourier transformation can be effective for strip noise [3,4,5]. These methods may not be able to detect bad streaks noise, because all information in the bad streaks is lost. Commonly used methods used for dealing with noise are divided into the following three categories: (1) multi-source; (2) single-source; and (3) hybrid [6].

In the first category, the noise gaps are filled by using multi-source data [6,7,8,9,10]. For instance, Ali used earlier Landsat images to repair noise and obtained an image of significantly higher quality [6]. Sun handled the Landsat images acquired from 2009 to 2010 by a localized linear histogram match and weighted liner regression [7]. Due to the shooting angle or the amount of radiation, there will be two unnatural lines beside the repair pattern. Due to the difficulty of multi-source information acquisition, this method is mainly used in an image covered by a wide range of noise.

In the second category, the noise is removed by a neighborhood pixel interpolator [11,12,13,14,15,16,17,18,19,20,21]. Han, using a traversal full map method that traverses the entire image, found the point pixel whose DN values were less than the two sides as bad pixels. Introducing a traversal searching method for abnormal pixel detection, the noise was replaced with the average of neighboring DN values [11,12]. However, the traversal method is time consuming and creates data redundancy. The threshold for identification, which represents the longest vertical feature on the ground, changes with each image, and is difficult define precisely. Gao used the neighborhood mean with median filter method to remove the noise in the Hyperion images [13]. Zhen improved the repair algorithm and used a Neighborhood Similar Pixel Interpolator (NSPI) for image repair [14]. Gao and Chen both aimed at improving the effect of repair, ignoring the problem that traversal full-map method would incur major costs in terms of time.

In the third category, the noise image is repaired by using the no noise image itself [22,23,24,25]. Maxwell used the coincident spectral data to fill the gap in the Landsat image [22]. Yu used the data from the same image in different bands to repair the bad streaks noise to enhance the effect of repair [23]. Screening images is a complex and time-consuming process, so this method is not suitable for large processing quantities.

However, by analyzing the bad streaks noise in the GF-1 images, it was found that their width is relatively narrow, and of limited range [26]. The single-source method is utilized, and the restoration method chosen is the Lagrange polynomial algorithm. The bad streaks noise, like the category 3 denoted by Tan [8] is discontinuous. As the start and end points of the bad streaks noise is in the same X coordinate, this paper presents a line tracking method, which can quickly and accurately distinguish bad streaks noise from the image. This method finds the bad streaks noise by line tracking method, and removes the noise by Lagrange polynomial algorithm, without influencing the image.

## 2. Data

In this paper, several gray-scale images with existing bad streaks from the GF-1, are chosen for repair.

**Figure 1:**
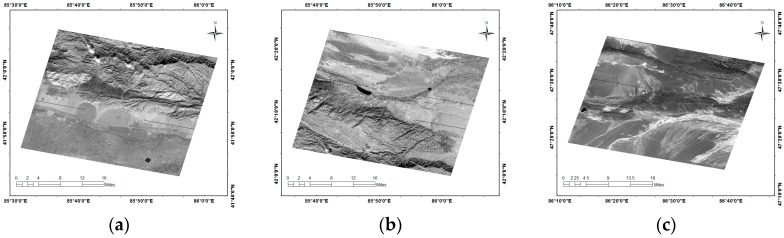
The original images of GF-1. (**a**) farmlands; (**b**) mountains; (**c**) islands.

As shown in Figure 1a, there are farmlands. The landform of the farmlands is relatively homogeneous. There are mountains in Figure 1b and islands in Figure 1c. These landforms are slightly more complex to verify the ability of an algorithm to repair different landforms. Specific data parameters are as shown in Table 1.

## 3. Methods

**Figure 2:**
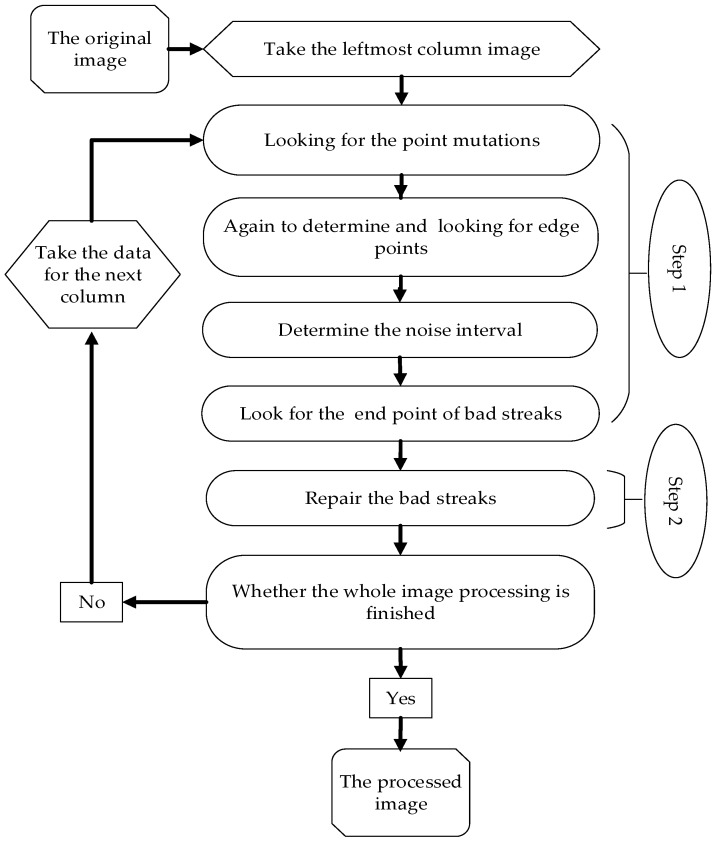
Steps of transverse stripe noise handling.

The restoration of corrupted image involves two steps. The first step is finding the coordinates of bad streaks noise with line tracing method. The following step shows a correction of bad streaks noise. In this paper, the overall flow chart of bad streaks removal is as shown in Figure 2.

### 3.1. Line Tracing Method

**Figure 3:**
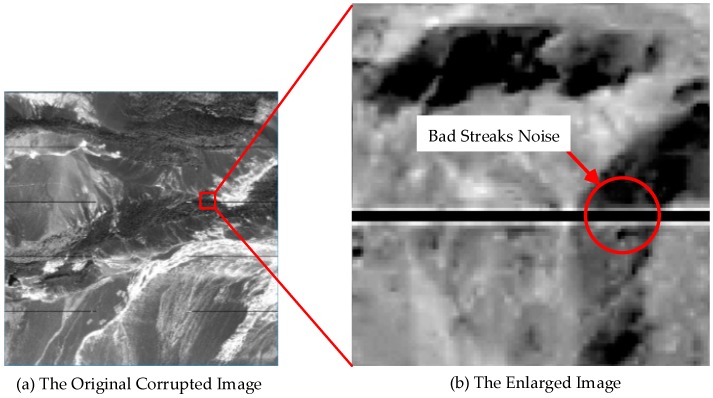
Image with bad streaks noise.

The basic principle of line tracing method is to locate the mutation that is caused by the bad streaks noise. As shown in Figure 3, the bad streaks noise is comprised of discontinuous, parallel, elongated lines, with start and end points in the same X coordinate. The DN value of pixels near the bad streaks noise will usually rise approximately 90, then rapidly decrease to zero. Therefore, the sudden reduction in the DN value is the mark of the edge. This point’s ordinate value can be considered as the noise edges. The last step is to find the start and end points in order to locate the extant of the bad streaks noise.

This method is divided into the several steps. The first step is to local the mutation point. Take the first column of image data, then each data minus the previous data to find the point where the DN value of the column data is mutated:

(1) $$|f(m_{1},t)-f(m_{1},t-1)|>T_{1}$$

where $m_{1}$ represents the position of the processing pixel abscissa, $f(m_{1},t)$ represents the DN values of pixels, and $T_{1}$ represents the threshold of the normal range of DN values. For example, this paper uses 1/3 of the mean of all DN values as the value of $T_{1}$. Value t represents the value of the y coordinate. By changing the value of t, all points will be verified by Equation (1).

If the point to be treated satisfies the Equation (1), the position will be marked as X point. In this way, $X_{1},X_{2},X_{3}\ldots$ points can be found. If there is no X point, re-take the abscissa data $m_{1}$ plus 10, and then repeat Step 1.

Step 2 outlines the detection of the bad streaks noise boundary. It should be noted that there are some points whose DN values change a lot and it will affect the results of Step 1. In order to avoid the original DN value mutation, these X points should be verified again. So some parallel points should be compared again.

(2) $$\begin{array}{l} |f(m_{1}+h,t)-f(m_{1}+h,t-1)|>T_{1}\\ |f(m_{1}+2h,t)-f(m_{1}+2h,t-1)|>T_{1}\\ \ldots \end{array}$$

where h indicates the size of the expected interference. This paper uses 10 as the value of h, and uses five points to verify the X. If there are all points greater than the threshold value, this point will be considered as the edge of bad streaks, and this point is marked as $A_{1}$. If X point cannot meet the requirements, this point will be discarded. After processing all X points, $A_{1}$, $A_{2}$, $A_{3}\ldots$ points will be found. If there is no A point, re-take the abscissa data $m_{1}$ plus 10, then repeat Step 1.

Step 3 determines the bad streak area. As the bad streaks are very slender, and the distance between the two bad streaks is large, comparing the distance of all A points can determine the bad streak area.

(3) $$\left|A_{n+1}-A_{n}\right|<T_{2}.$$

If Equation (3) is satisfied, the area between the $A_{n}$ and $A_{n+1}$ is marked as bad streaks. The threshold $T_{2}$ here must be slightly greater than the value of the maximum width of the bad streaks. The value used in this paper is 10. If the A point is very close to the edge of the image, the area between A and the edge of the image also is marked as a bad streak.

Step 4 relates to the search for the horizontal of the end point. Then only the points which have the same ordinate value with the A points should be analyzed instead of all points within the image.

(4) $$|f(i,A_{n})-f(i,A_{n}-1)|>T_{1}$$

where *i* represents the value of the x coordinate and the initial value of *i* is $m_{1}$. If the data point satisfies Equation (4), it is still located within an area of bad streaks. So the abscissa i will increase by one, and this new point will be compared again until it no longer satisfies Equation (4). The penultimate point is the end of the bad streaks area, and it is marked as K.

In Step 5, a horizontal axis is added and the process is looped until K reaches the image edge.

After these steps, many rectangles are found, and only the problematic pixels in the rectangles require further repair.

### 3.2. Lagrange Polynomial Algorithm

Since the information of the bad streaks area is completely missing, the data in this region has to be simulated to make the image more suitable for observation. In this study, the Lagrange polynomial algorithm is chosen. The principle of the Lagrange polynomial algorithm is that Lagrange interpolation algorithm [27] can build a function by n points, and use this function to speculate the remaining value of the information of the originally unknown points.

Now that bad streaks on the image are very slender compared with the whole image, the pixels between the bad streaks are interrelated. The Lagrange interpolation algorithm is used to construct a function that passes both the top and the bottom of the bad streaks. Then, the information on the bad streaks position can be repaired by information obtained from the function value information. Specific algorithm steps are as follows:

In general, an n-degree function, b=f(a), should be constructed to pass n + 1 points, $(a_{0},b_{0}),$ $(a_{1},b_{1}),$ $\left(a_{2},b_{2})\ldots (a_{n},b_{n}\right)$:

(5) $$\mathrm{Pn}(a_{k})=b_{k},\;k=0,1,\ldots ,n$$

$a_{k}$ represents the abscissa position of the point to be passed, and $b_{k}$ represents the magnitude of the value of the response through the point. In order to estimate the value of the point on f(a), the value of the point on Pn(a) can be used as a reference.

In the remote sensing image, the original DN value can be located on the same function line as the upper and lower points:

(6) $$p_{j}(a)=\prod_{i\in I_{j}}\frac{a-a_{i}}{a_{j}-a_{i}}$$

$p_{j}(a)$ is a (n-1)-degree function, and $\forall i\in I_{j},\;p_{j}(a_{i})=0$&$p_{j}(a_{j})=1$.

Resulting in a new equation:

(7) $$L_{n}(a)=\sum_{j=1}^{n}b_{j}p_{j}(a)$$

$L_{n}(a)$ indicates a curve that can pass through these n points.

Then, the two points above the streaks and the two points below the streaks are selected as the reference data, so the required transformation equation shows:

(8) $$\begin{array}{l} f(x)\\ =\frac{\left(x-x_{2})(x-x_{3})(x-x_{4}\right)}{\left(x_{1}-x_{2})(x_{1}-x_{3})(x_{1}-x_{4}\right)}y_{1}\\ +\frac{\left(x-x_{1})(x-x_{3})(x-x_{4}\right)}{\left(x_{2}-x_{1})(x_{2}-x_{3})(x_{2}-x_{4}\right)}y_{2}\\ +\frac{\left(x-x_{1})(x-x_{2})(x-x_{4}\right)}{\left(x_{3}-x_{1})(x_{3}-x_{2})(x_{1}-x_{4}\right)}y_{3}\\ +\frac{\left(x-x_{1})(x-x_{2})(x-x_{3}\right)}{\left(x_{4}-x_{1})(x_{4}-x_{2})(x_{4}-x_{3}\right)}y_{4} \end{array}$$

where $x_{1},$ $x_{2},$ $x_{3},$ $x_{4}$ represent the ordinates of the selected four reference points; $y_{1},$ $y_{2},$ $y_{3},$ $y_{4}$ are the DN value corresponding to the reference points; x is the ordinate position of the point to be repaired; the value of f(x) is the DN value after repair.

**Figure 4:**
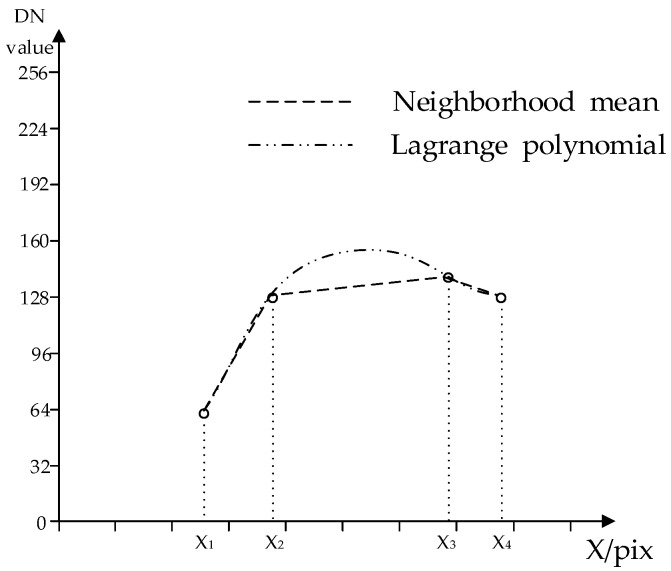
Compare with neighborhood mean and Lagrange polynomial algorithm.

As shown in Figure 4, in the coordinate system two ways were used to fit the curve, and it shows that the Lagrange polynomial is smoother than the neighborhood mean value. By using this equation, the existing information in the image can be used to repair the radiation value information of the bad streaks.

### 3.3. Validation

The verification of the method consists of two parts. The first part is the quantitative analysis of the line tracing method, by comparing the detection accuracy and detection efficiency. The second part is the qualitative and quantitative analysis of the Lagrange polynomial algorithm. The repaired images must be able to link the nearby images and be suitable for observation. So the qualitative analysis is performed by visual observation. This paper introduces the image entropy as the judgment index for quantitative analysis. Image entropy is an important feature of the amount of data information, and the calculation equation is as follows:

(9) $$H=-\sum_{i=0}^{N}p_{i}\log_{2}(p_{i})$$

where *H* represents the image entropy and $p_{i}$ represents the probability that each DN value appears. *N* indicates the maximum DN value in the images. The greater the image entropy, the greater the amount of information on the image.

## 4. Results

### 4.1. Results of Line Tracing Method

By comparing the pixels in the original image affected by the bad streaks noise with the pixels calculated by the line tracing method and traversal full-map method, there are some differences between the two methods (Table 2).

In this study, the total number of pixels affected by bad streaks noise is 355,100. The number of points found by line tracing method is 352,925. The number of points that are not found is 2175, which is less than that of the 2873 using the traversal full-map method. In many experiments, the leak detection rate remained at 1.0% or less, and false detection rate remained at 0.1% or less, with a high degree of detection accuracy maintained.

Therefore, the line tracing method can accurately find the position of the transverse fringe noise by the abrupt change of the DN value in the image. This method also judges the point affected by the bad streaks noise, and improve the detection accuracy compared with the traversal full map method.

The detection efficiency was calculated with a computer equipped with dual-core CPU i5 memory 8G, and the data processing software platform is MATLAB.

Repetition of this experiment using other images, found that the test results were basically the same, so the specific data in this article is not repeatedly listed. Compared with the traversal full-map method, the line tracing method can shorten the time required to complete image repairs by half, and can improve the image processing efficiency, as shown in Table 3.

### 4.2. Results of Lagrange Polynomial Algorithm

**Figure 5:**
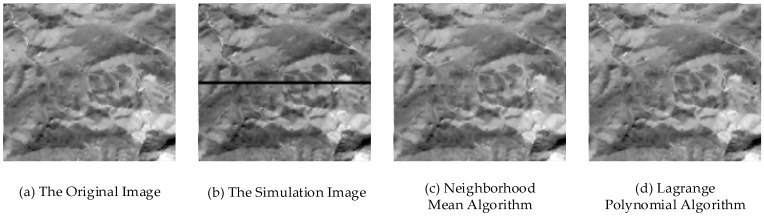
The results of repair algorithm on the simulation image.

A simulation image is used for this experiment. The DN value of a part of the pixel in the original data was changed into zero, and bad streaks were added to obtain the simulation image (Figure 5a,b). The neighborhood mean algorithm and the Lagrange polynomial algorithm are tested on the simulation image, and the results are shown in Figure 5c,d. The image entropy is calculated for the area of the bad streaks. The results are shown in Table 4.

First, by observing the image, it is found that the bad streaks are removed and the image becomes suitable for observation, and the boundaries of bad streaks are relatively insignificant. Then, by comparing the image entropy, it is found that both of the algorithms can restore the value of the image entropy to approximately the original data. The result of the Lagrange polynomial algorithm is better than the neighborhood mean algorithm. The simulation experiment shows that the two algorithms are feasible in removing noise.

**Figure 6:**
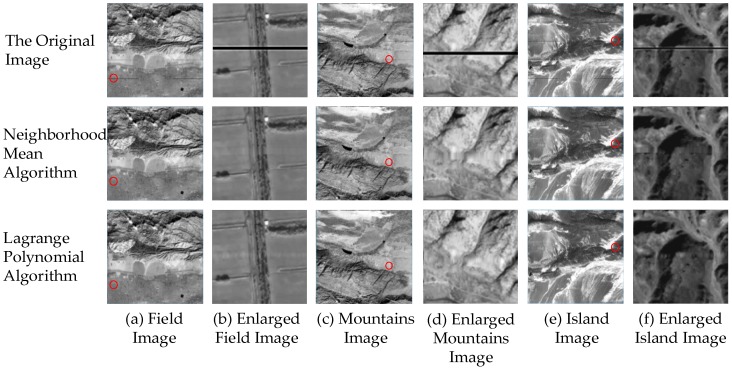
The results of repair algorithm on the real GF-1 images.

The next phase involves verifying the algorithm using real GF-1 images. Through the repair and comparison of the bad streaks noise in different areas, it is judged whether the algorithm can effectively make the image smoother and easier to observe in the area of bad streaks noise. As CCD cameras are instant cameras, there will not be the original undisturbed images to compare. The image entropy is continuous within the data area, so the image entropy of the adjacent area with the same area can be used for comparison. After experimenting with different images, the following three images are taken as examples. The experimental results are shown in Figure 6 and Table 5.

First, qualitative analysis is conducted through direct observation. In contrast to the overall image, as shown in Figure 6a,c,e, the two algorithms can basically remove the noise. However, by enlarging the image of the noise region, it is found that, in the homogeneous landform, the two algorithm effects are basically flat. The repair effect of the Lagrange polynomial algorithm is obviously better than the neighborhood mean algorithm in the region where the geographical environment is more complex, as in Figure 6d,f. The boundaries of the repaired image by the Lagrange interpolation are relatively more insignificant than the neighborhood mean algorithm. Then, there is the quantitative analysis by comparing the image entropy. It is found that the image entropy of the repaired images by the Lagrange polynomial algorithm is more accurate when compared to the original undisturbed images than the neighborhood mean algorithm.

## 5. Discussion

In this paper, the results of the line tracing detection method are discussed. By finding the location of the bad streaks noise, although the complexity in estimating the bad streaks noise is increased, the line tracing method also greatly reduces the number of times to estimate the non-noise data points. This ultimately reduces the time spent searching for the noise region. In terms of accuracy, the algorithm does not improve much. This algorithm can determine nearly all of the points, even the point whose DN values changes subtly or abnormally increases, which is frequently missed by the traversal full map method.

As for the repair effect, the Lagrange polynomial algorithm used in this paper can effectively recover the information of the points in the bad streaks, while preserving the original image information. Since the Lagrange polynomial method fits a curve rather than a straight line approximating the neighborhood mean method, the effect of the repair is more in line with people's observation habits than the neighborhood mean method. Therefore, Lagrange polynomial method is suitable for bad streaks noise.

## 6. Conclusions

Aiming at the characteristics of bad streaks, this paper proposes an improved algorithm for removing bad line streaks in the image by the line tracing method and Lagrange polynomial algorithm. The line tracing method in the algorithm can find out the position of the bad streaks by the DN value mutation caused by the bad streaks, and the Lagrange polynomial algorithm can effectively utilize the inheritance of the DN value in the linear array image. This allows bad streaks to be repaired without the interference from the new noise. In a word, the accuracy of the full-map method is improved, and the efficiency is improved as well as the effect of the repair of the bad streaks compared with the neighborhood mean method.

## Figures and Tables

**Table 1:** Overview of test datasets.

ID	Lat/Long	Terrain	Sensor	Image Size	Color Depth (bit)	Resolution (m)
a	85.8/41.9	Field	PAN	18,192 × 18,000	16	2.0
b	85.8/42.2	Mountains	PAN	18,192 × 18,000	16	2.0
c	86.5/47.4	Island	PAN	18,192 × 18,000	16	2.0

**Table 2:** Detection result statistics of the bad streaks noise pixel point.

Method	The Number of Found	The Number of Not Found	The Number of Wrong
Traversal full map method	352,277	2823	0
Line tracing method	352,925	2175	0

**Table 3:** Comparison of time used in the algorithm.

Method	Time (s)
Traversal full map method	61.1
Line tracing method	26.9

**Table 4:** The image entropy of repaired images.

Method	The Original Image	The Simulation Image	Neighborhood Mean Algorithm	Lagrange Polynomial Algorithm
Image Entropy	6.2520	0	6.1839	6.2897

**Table 5:** The image entropy of different areas.

Terrain	The Original Image	Neighborhood Mean Algorithm	Lagrange Polynomial Algorithm
Field	5.6661	5.5858	5.6841
Mountains	8.0018	7.7176	8.0883
Island	6.8297	6.5766	6.9501
